# Herbal Medicines for the Treatment of Active Ulcerative Colitis: A Systematic Review and Meta-Analysis

**DOI:** 10.3390/nu16070934

**Published:** 2024-03-23

**Authors:** Preetha Iyengar, Gala Godoy-Brewer, Isha Maniyar, Jacob White, Laura Maas, Alyssa M. Parian, Berkeley Limketkai

**Affiliations:** 1 Department of Medicine, University of California Los Angeles David Geffen School of Medicine, Los Angeles, CA 90095, USA; piyengar@mednet.ucla.edu; 2 Department of Medicine, University of Miami, Miami, FL 33136, USA; galagodoyb@gmail.com; 3 Center for Inflammatory Bowel Diseases, Vatche and Tamar Manoukian Division of Digestive Diseases, University of California Los Angeles David Geffen School of Medicine, Los Angeles, CA 90095, USA; ishamaniyar2019@gmail.com; 4 Welch Library, Johns Hopkins University School of Medicine, Baltimore, MD 21287, USA; jwhit191@jhmi.edu; 5 Department of Medicine, Johns Hopkins University School of Medicine, Baltimore, MD 21287, USA; lmaas3@jhmi.edu; 6 Division of Gastroenterology and Hepatology, Johns Hopkins University School of Medicine, Baltimore, MD 21287, USA; aparian1@jhmi.edu

**Keywords:** herbal medicines, plant extracts, phytotherapy, dietary supplements, ulcerative colitis, complementary therapies, integrative medicine

## Abstract

Herbal medicines are used by patients with IBD despite limited evidence. We present a systematic review and meta-analysis of randomized controlled trials (RCTs) investigating treatment with herbal medicines in active ulcerative colitis (UC). A search query designed by a library informationist was used to identify potential articles for inclusion. Articles were screened and data were extracted by at least two investigators. Outcomes of interest included clinical response, clinical remission, endoscopic response, endoscopic remission, and safety. We identified 28 RCTs for 18 herbs. In pooled analyses, when compared with placebo, clinical response rates were significantly higher for *Indigo naturalis* (IN) (RR 3.70, 95% CI 1.97–6.95), but not for *Curcuma longa* (CL) (RR 1.60, 95% CI 0.99–2.58) or *Andrographis paniculata* (AP) (RR 0.95, 95% CI 0.71–1.26). There was a significantly higher rate of clinical remission for CL (RR 2.58, 95% CI 1.18–5.63), but not for AP (RR 1.31, 95% CI 0.86–2.01). Higher rates of endoscopic response (RR 1.56, 95% CI 1.08–2.26) and remission (RR 19.37, 95% CI 2.71–138.42) were significant for CL. CL has evidence supporting its use as an adjuvant therapy in active UC. Research with larger scale and well-designed RCTs, manufacturing regulations, and education are needed.

## 1. Introduction

Ulcerative colitis (UC) is an immune-mediated chronic inflammatory bowel disease (IBD) characterized by relapsing and remitting mucosal inflammation in the colon [1,2,3]. The worldwide prevalence of UC is on the rise and patients often experience debilitating symptoms including diarrhea, abdominal pain, rectal bleeding, extra-intestinal manifestations, and an increased risk of colorectal cancer [1,2,4]. The complex pathophysiology of UC has been attributed to a combination of genetic and environmental factors that result in immune system dysregulation, including impaired mucin synthesis, increased activation of inflammatory cytokines, regulatory and effector T cell imbalance, and gut microbial dysbiosis [1,5]. Current treatment strategies target inflammation including aminosalicylates, immunosuppressants, and biologics with the goal of improving clinical symptoms and inducing remission [2,5]. Despite these therapies, around 15% of patients will continue to struggle with relapsing disease and will eventually require partial or total colectomies [4]. Immunosuppressant medications also carry the risk of serious adverse events, including malignancies, such as lymphoma, and infections, such as the reactivation of tuberculosis [3]. There continues to be an unmet need for effective and tolerable therapies.

Due to a perceived lack of response to standard therapy, concerns about the side effects of conventional therapies, and a feeling of control over their disease many patients with IBD turn towards complementary and alternative medicine (CAM) more so than the general population [3,5,6,7,8,9]. Herbal medicines are the most used CAM approach with 19–54% of patients with IBD reporting their use [7,10,11,12]. However, many patients do not disclose herbal medicine use with their providers and many providers lack the knowledge and access to high quality clinical data to be able to guide their patients with regard to CAM use [6,7,8,13]. 

Although there have been narrative and systematic reviews of dietary supplements and herbal medicines for IBD [14,15,16,17,18,19], many of these reviews have focused only on the most commonly used supplements, included observational studies, and included patients with both active disease and remission. The aim of this study was to perform a comprehensive systematic review and meta-analysis of randomized controlled trials (RCTs) of herbal medicines used in the treatment of active UC. 

## 2. Materials and Methods

**Figure 1:**
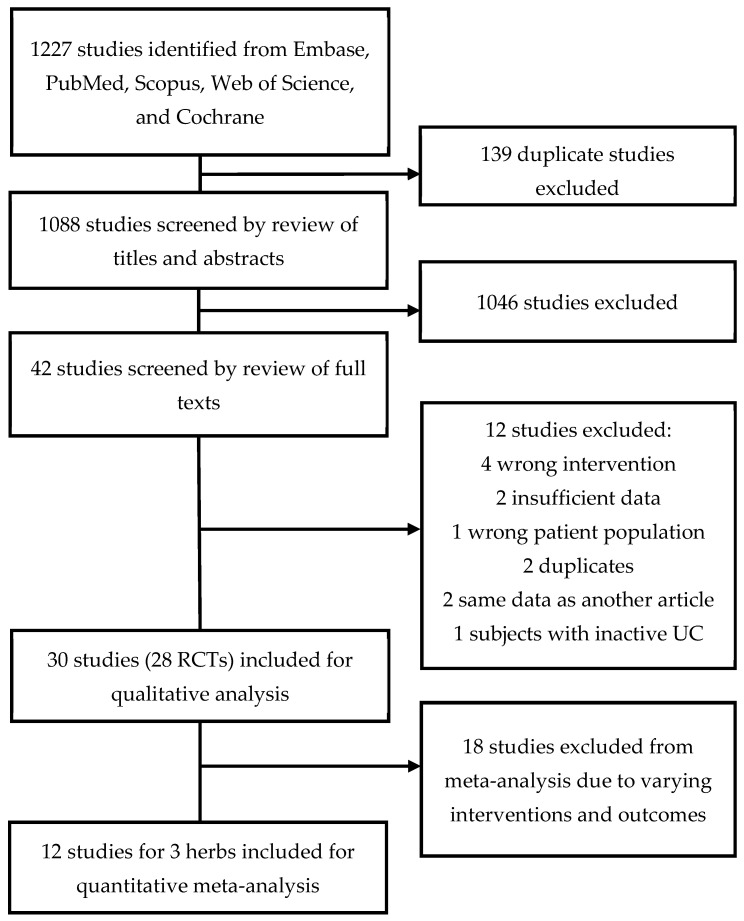
PRISMA flow diagram.

This systematic review was conducted using PRISMA guidelines (PRISMA checklist available in Supplementary Materials) but was not registered. Candidate studies for inclusion into this review were identified through systematic literature searches from inception to September 2022 in the following electronic databases: Medline (PubMed), EMBASE (Embase.com), the Cochrane Library (Cochrane Database of Systematic Reviews, Cochrane Central Register of Controlled Trials (CENTRAL), Cochrane Methodology Register, and Web of Science (Figure 1). The search strategy was developed and executed by a library informationist. Keywords and appropriate controlled vocabulary terms, including the following Emtree terms: “ulcerative colitis”, “medicinal plant”, “plant extract”, “plant medicinal product”, and “randomized controlled trial” were used to develop the search strategies for all databases. Studies were considered eligible if they met the following criteria: (1) human patients with active mild, moderate, or severe UC at the time of enrollment, as defined by the study; (2) prospective controlled studies controlled either with placebo or conventional treatment; and (3) intervention included an herbal medicine. Studies were excluded for the following criteria: (1) they were performed in animals, (2) IBD was in remission at baseline, (3) studies were biochemical models, case series, case reports, narrative reviews, or editorials, and (4) the intervention combined multiple herbal ingredients. The primary outcomes were clinical remission and clinical response. Secondary outcomes included endoscopic response, endoscopic remission, and safety. 

All titles, abstracts, and full-length publications of selected articles were screened for final inclusion by two independent reviewers (Figure 1). A third investigator served to adjudicate any discrepancies at all stages of study selection. Clarification regarding the potential republishing of data between poster abstracts and full-length publications was obtained by contacting the authors as needed. The data variables extracted from articles are presented in Table 1. The Cochrane Risk of Bias tool was used to assess the potential risk of bias in each included study. The Grading of Recommendations Assessment, Development, and Evaluation (GRADE) framework was used to assess the quality of evidence for each outcome.

Studies were grouped by herbal intervention. Meta-analyses with Forest plots were generated with pooled studies that shared similar comparators and outcomes. Risk ratio (RR) and 95% confidence intervals (CIs) were estimated while applying random effects models. For each outcome, heterogeneity was qualitatively and quantitatively assessed, the latter using the I^2^ statistic and Χ^2^ test with *p* < 0.10 considered significant heterogeneity. A two-tailed α threshold of 0.05 was otherwise used to delineate statistical significance. Analyses were conducted using Review Manager 5.4.1 (The Cochrane Collaboration, Oxford, UK).

## 3. Results

The comprehensive literature review identified 1227 studies, which underwent selection and review by the authors, as detailed in Figure 1. Reasons for excluding articles included inappropriate intervention, insufficient data, wrong patient population, or duplicate studies. Table 1 outlines the general characteristics of the studies included. Table 2 summarizes the risk of bias for each study.

**Table 1:** Summary of articles identified in systematic review.

Herb	Author/ Year	Country	Baseline Disease Activity	Number of Patients Randomized	Control	Intervention	Use of Concomitant UC Medications	Treatment Duration	Select Results at End of Study (Intervention vs. Control)	Adverse Events
*Curcuma longa*	Banerjee et al., 2021 [20]	India	Mild–moderate UC	69	Placebo with oral and rectal mesalamine	50 mg bioenhanced curcumin PO BID with oral and rectal mesalamine	Patients were biologic and immunomodulator naive	6 weeks, 3 months	(1) Clinical response: 52.9% vs. 14.3% (*p* = 0.001) at 6 weeks; 58.8% vs. 28.6% at 3 months (*p* = 0.013) (2) Clinical remission): 44.1% vs. 0% (*p* < 0.01) at 6 weeks; 55.9% vs. 5.7% at 3 months (*p* < 0.01) (3) Endoscopic remission: 35.3% vs. 0% (*p* < 0.001) at 6 weeks; 44% vs. 5.7% at 3 months (*p* < 0.001)	No difference in mild (i.e., abdominal bloating) or severe AEs
	Kedia et al., 2017 [21]	India	Mild–moderate UC	62	Placebo with oral mesalamine	150 mg purified curcumin capsules PO TID with oral mesalamine	6.5% used AZA	8 weeks	(1) Clinical response: 20.7% vs. 36.4% (*p* = 0.18) (2) Clinical remission: 31.3% vs. 27.3% (*p* = 0.75) (3) Mucosal healing: 34.5% vs. 30.3% (*p* = 0.72)	No difference in mild (i.e., self-limited arthralgias) AEs
	Kumar et al., 2018 [22]	India	Mild–severe UC	53	Placebo powder QD with oral mesalamine	10 g/d PO *C. longa* powder QD *with* 2.4 g/d PO mesalamine	Not disclosed	8 weeks	(1) Clinical response: 60.7% vs. 52% (*p* = 0.412) (2) Reduction in fecal calprotectin by ≥25 units: 83.3% vs. 50% (*p* = 0.034)	No difference between groups
	Lang et al., 2015 [23]	Israel	Mild–moderate UC	50	Placebo with oral and enema/suppository mesalamine	1.5 g curcumin capsules PO BID with oral and enema/suppository mesalamine	Immunomodulators (AZA, 6-MP) allowed if stable dose. No recent use of steroids, cyclosporine, or anti-TNFα agents permitted.	4 weeks	(1) Clinical response: 65.3% vs. 12.5% (*p* < 0.001) (2) Clinical remission: 53.8% vs. 0% (*p* = 0.01) (3) Endoscopic response: 45.4% vs. 0% (*p* < 0.043) (4) Endoscopic remission: 38% vs. 0% (*p* = 0.04)	No difference between groups in mild (nausea, increased stool frequency, bloating) or severe (UC flair, peptic ulcer) AEs
	Masoodi et al., 2018 [24]	Iran	Mild–moderate UC	56	Placebo with oral mesalamine	80 mg curcuminoids nanomicelles PO TID with oral mesalamine	Topical mesalamine, prednisolone, azathioprine, or TNFα-inhibitors allowed.	4 weeks	(1) Clinical symptoms: Difference between groups in urgency of defecation score (*p* = 0.041), but not in number of daily bowel movements (*p* = 0.13), blood in stool (*p* = 0.781), or nocturnal bowel movements (*p* = 0.131) (2) Mean SCCAI: 1.71 ± 1.84 vs. 2.68 ± 2.09 (*p* = 0.050)	No significant difference between mild (flatulence, dyspepsia, headache, increased headache, nausea, yellow stool) AEs
	Sadeghi et al., 2020 [25]	Iran	Mild–moderate UC	70	Placebo capsules	500 mg curcumin capsules PO TID with meals	Concomitant salicylates, immunomodulators, or steroids allowed; cannot be on TNFα inhibitors	8 weeks	(1) Clinical response: 93.5% vs. 59.4% (*p* < 0.001) (2) Clinical remission: 83.9% vs. 43.8% (*p* = 0.001) (3) Change in IBDQ-9: 9.5 ± 8.4 (*p* = 0.001) vs. 4.09 ± 7.7 (*p* = 0.004)	Mild AEs reported (skin allergy, dyspepsia, heartburn)
	Shivakumar et al., 2011 [26]	India	Active UC	53	Placebo powder PO QD + mesalamine/steroids	10 g curcumin powder QD + mesalamine/steroids	Not disclosed	8 weeks	(1) Reduction in Mayo score: 0.56 ± 0.71 vs. 0.43 ± 0.78 (*p* = 0.56) (2) Decrease of 1 point in histological activity score: 62.5% vs. 43.47% (*p* = 0.19) (3) Fecal calprotectin levels: 175.22 ± 179.56 vs. 65.19 ± 240.57 μg/g (*p* = 0.001)	Not reported
	Singla, 2014 [27]	India	Mild to moderate distal UC	45	Placebo enema with oral mesalamine	140 mg NCB-02 enema QHS with oral mesalamine	Steroids, 5-ASA, AZA	8 weeks	(1) Clinical response: 56.5% vs. 36.4% (*p* = 0.175) (2) Clinical remission: 43.4% vs. 22.7% (*p* = 0.14) (3) Endoscopic response: 52.2% vs. 36.4% (*p* = 0.29)	No difference in severe AEs (UC flare)
*Indigo naturalis*	Naganuma et al., 2018 [28]	Japan	Moderate UC	86	Placebo capsules	4250 mg, 125 mg, or 62.5 mg in each capsule IN powder capsules PO BID (total 0.5, 1, or 2 g/d)	Steroids, thiopurines, TNFα inhibitors	8 weeks	(1) Clinical response: 0.5 g IN 69.6% (*p* = 0.002); 1 g 75% (*p* = 0.0001); 2 g IN 81% (*p* < 0.0001) vs. 13.6% (2) Clinical remission: 0.5 g IN 26.1% (*p* = 0.0959); 1 g IN 55% (*p* = 0.0004); 2 g IN 38.1% (*p* = 0.0093) vs. 4.5% (3) Endoscopic response: 0.5 g IN 56.5% (*p* < 0.0045); 1 g IN 60% (*p* < 0.0032); 2 g IN 47.6% (*p* < 0.0217) vs. 13.6%	Mild AEs included liver dysfunction, headache, epigastric/abdominal pain, nausea
	Uchiyama et al., 2020 [29]	Japan	Mild–moderate UC	46	500 mg rice starch capsule PO BID	500 mg IN powder capsules PO BID	5-ASA, prednisolone, AZA, biologics	2 weeks	(1) Clinical response: 82.6% vs. 26.3% (*p* = 0.0003) (2) Marked clinical response: 60.9% vs. 5.3% (*p* = 0.0002)	Mild AEs included headache, constipation, palpitations
*Andrographis paniculata*	Sandborn et al., 2013 [30]	USA Canada Germany Romania Ukraine	Mild to moderate UC	223	Placebo capsules	400 mg or 600 mg capsules containing *A. paniculata* ethanol extract (HMPL-004) PO TID (total 1.2 g or 1.8 g/d)	Concomitant mesalamine, sulfasalazine, balsalazide, or olsalazine. Subjects with use of other UC meds within 6 weeks were excluded.	8 weeks	(1) Clinical response: 1200 mg 44.6%, (*p* = 0.5924) 1800 mg 59.5% (*p* = 0.0183), combined 1200 mg + 1800 mg 52% (*p* = 0.0465) vs. 40% (2) Clinical remission: 1200 mg 33.8% (*p* = 0.2718), 1800 mg 37.8% (*p* = 0.1011), 1200 mg + 1800 mg 35.8% (*p* = 0.2516) vs. 25.3% (3) Endoscopic response: 1200 mg 37.8% (*p* = 0.5281), 1800 mg 50% (*p* = 0.0404), 1200 mg + 1800 mg 43.95% (*p* = 0.1025) vs. 33.3%	No significant difference in mild to moderate (rash, abdominal pain, diarrhea, dyspepsia, AST/Alk Phos, GGT elevation) or severe AEs
	Tang et al., 2013 [31]	China	Mild to moderate UC	125	Oral mesalazine	400 mg capsules containing *A. paniculata* ethanol extract (HMPL-004) PO TID (total 1.2 g/d)	No other concomitant UC medications permitted, but previous treatment with 5-ASA and/or steroids permitted	8 weeks	(1) Clinical remission: 21% vs. 16% (2) Partial clinical remission: 36% vs. 36% (3) Clinical improvement: 19% vs. 29% (4) Endoscopic response: 26% vs. 29% (4) Endoscopic remission: 28% vs. 24% (5) Histologic improvement: 53% vs. 40% (*p* < 0.001)	13% int. vs. 27% cont. had ≥1 AE. Mild to moderate AEs included aphthous ulcer, abdominal pain, bloody stools, rash, hematuria, fevers, WBC decrease, and diarrhea among others. 4% int. vs. 0% cont. had severe AEs
*Aloe vera*	Langmead et al., 2004 [32]	England	Mild to moderate UC	44	Placebo liquid	100 mL *Aloe vera* gel PO BID	Concomitant 5-ASA, AZA, topical steroids, or none	4 weeks	(1) Clinical remission: 30% vs. 7% (*p* = 0.09) (2) Clinical response: 47% vs. 14% (*p* = 0.048) (3) Sigmoidoscopic remission: 27% vs. 18% (*p* = 0.69) (4) Histological remission: 29% vs. 44% (*p* = 0.43)	No difference between groups in mild AEs (abdominal bloating, foot pain, sore throat, ankle swelling, acne, eczema)
	Pica et al., 2021 [33]	Italy	Mild to moderate active ulcerative proctosigmoiditis	44	Placebo enema with oral mesalazine	60 mL *Aloe vera* gel enema QD	Not reported	4 weeks	(1) Change in average DAI: 6.66 ± 1.75 to 3.27 ± 2.07 (*p* = 0.002) vs. 6.19 ± 1.63 to 5.90 ± 2.16 (*p* = 0.780)	Not reported
*Arthrospira platensis*	Moradi et al., 2021 [34]	Iran, UK	Mild to moderate UC	80	Placebo capsules	500 mg Spirulina capsule PO BID before lunch and dinner (total 1 g/d)	Oral or rectal mesalazine, sulfasalazine, prednisolone, AZA	8 weeks	(1) Anthropometric parameters: No significant difference in body weight, neck circumference (NC), hip circumference (HC), waist circumference (WC), waist to hip ratio (WHR), body mass index (BMI), or blood pressure within each group. (2) Sleep quality: Significant decrease in sleep disturbances (*p* = 0.004) and sleep quality (*p* = 0.01) in each group per PSQI over study period. (3) Mood, stress, quality of Life: Significant decrease in stress (*p* < 0.001 vs. *p* = 0.04) and depression (*p* = 0.01 vs. *p* = 0.02) within each group over study period. Increase in SIBDQ (*p* < 0.001 vs. *p* = 0.01) within each group. Significant difference in stress score (*p* = 0.04) and quality of life (*p* = 0.03) between groups.	Mild bloating
*Boswellia serrata*	Gupta et al., 1997 [35]	India	Mild to moderate active UC	30	Oral sulfasalazine	300 mg encapsulated powdered gum resin of *B. serrata* PO TID	Not permitted to take other drugs	6 weeks	(1) Clinical remission: 82.4% vs. 75% (OR 0.673, *p* = 1) (2) Sigmoidoscopic improvement from grade III to grade 0-I: 75% vs. 75% (OR 0.740, *p* = 1)	Mild AEs including retrosternal burning, nausea, fullness of abdomen, epigastric pain, anorexia in treatment group
Green tea	Dryden et al., 2013 [36]	USA	Mild to moderate UC	20	Placebo capsules	Cohort 1: 200 mg Polyphenon E capsule + 1 placebo capsule PO BID Cohort 2: Two 200 mg Polyphenon E capsules PO BID (total 400 mg/d)	Concomitant 5-ASA, AZA, 6-MP allowed; steroids and other immunosuppressants not permitted	8 weeks	(1) Clinical response: 66.7% vs. 0% (*p* = 0.03) (2) Clinical remission: 53.3% vs. 0% (*p* = 0.10)	No significant difference in mild to moderate AEs (heartburn, bloating, flatulence, headache, diarrhea, increased thirst) One patient in the treatment group required hospitalization for *C. difficile* infection
Flaxseed	Morshedzadeh et al., 2019 [37]	Iran	Mild to moderate UC	90	Medical advice and routine medications	15 g ground flaxseed (GF) mixed in cold water BID *or* 10 g flaxseed oil (FO) QD	Not permitted to be taking concomitant steroids, AZA, 6-MP, methotrexate, cyclosporine, TNFα inhibitors	12 weeks	(1) Mayo score: 3.66 (GF) and 3.78 (FO) vs. 4.90 (*p* = 0.006) (2) IBDQ score: 48.96 (GF) and 48.08 (FO) vs. 42.08 (*p* < 0.001) (3) Fecal calprotectin (µg/mg): 424.20 (GF) and 484.20 (FO) vs. 602.32 (*p* = 0.008)	None reported
	Morshedzadeh et al., 2021 [38]	Iran	Mild to moderate UC	90	Routine treatment protocol	15 g ground flaxseed (GF) mixed in cold water BID *or* 10 g flaxseed oil (FO) QD	Not permitted to be taking concomitant steroids, AZA, 6-MP, methotrexate, cyclosporine, TNFα inhibitors	12 weeks	(1) IL-10 serum levels (pg/dL): 51.29 (GF) and 47.47 (FO) vs. 40.49 (*p* = 0.002) (2) hs-CRP (mg/L): 4.06 (GF) and 4.00 (FO) vs. 3.8 (*p* < 0.001)	None reported
Licorice	Sun et al., 2018 [39]	China	Active UC	94	Mesalazine enteric-coated tablets	Licorice decoction combined with mesalazine	Mesalazine	6 weeks	(1) Clinical total effective rate: significantly higher in int. vs. cont. (*p* < 0.05) (2) Levels of serum IL-6, IL-17, and TNFα: significantly lower in int. vs. cont. (*p* < 0.05) (3) IBDQ scores: significantly higher in int. vs. cont. (*p* < 0.05)	None reported
Olive oil	Morivaridi et al., 2020 [40]	Iran	Mild to severe UC and remission	40	50 mL canola oil (CO) PO QD	50 mL extra virgin olive oil (EVOO) PO QD	Mesalazine, prednisolone, azathioprine, others	20 days of EVOO or CO + 14 days of washout + 20 days of EVOO or CO	(1) Inflammatory markers: Change in mean ESR (−1.18 ± 7.00 vs. 1.87 ± 8.10; *p* = 0.03); change in mean hs-CRP (−1.31 ± 1.74 vs. 0.36 ± 1.15; *p* < 0.001); change in TNFα (−3.92 ± 19.33 vs. 8.16 ± 84.13; *p* = 0.37) (2) Clinical symptoms: GSRS score decreased significantly in EVOO group (*p* < 0.05); bloating (*p* = 0.04), constipation (*p* < 0.001), fecal urgency (*p* < 0.001), incomplete defecation (*p* = 0.04) decreased significantly with EVOO. Change in Mayo score not significant.	None reported
*Pistacia lentiscus*	Papada et al., 2018 [41]	Greece, UK, Serbia	Mild to moderate UC	20	Placebo tablets	Four 700 mg tablets containing 70% *Pistacia lentiscus* (PL) PO QD (total 2.8 g/d)	Mesalazine, AZA, steroids	3 months	(1) Change in oxidative stress markers: No significant differences between groups. OxLDL: −18.4 ± 46 vs. −10.7 ± 58.8; OxLDL/HDL: −1.03 ± 1.96 vs. 0.43 ± 1.83; OxLDL/LDL: −0.39 ± 0.76 vs. −0.27 ± 0.92 (2) Change in amino acids levels: Significant differences between groups in leucine −23.8 nmoL/mL vs. 16.1 nmoL/mL (*p* = 0.043), serine 13.5 nmoL/mL vs. −16.3 nmoL/mL (*p* = 0.028), and glutamine 50.3 nmoL/mL vs. −37.5 nmoL/mL (*p* = 0.038)	None reported
*Plantago major*	Baghizadeh et al., 2021 [42]	Iran	Mild–severe UC and remission	61	Two roasted wheat flour capsules PO TID before meals	Two 600 mg *p. major* seed capsules PO TID before meals (total of 3600 mg/day)	Continuation of routine drugs	8 weeks	(1) Clinical response: 5.21 ± 3.91 to 2.43 ± 2.71 vs. 4.00 ± 3.81 to 2.09 ± 3.01 (*p* = 0.282) (2) % subjects with gastrointestinal symptoms: gastroesophageal reflux 32% to 11% vs. 26% to 22% (*p* = 0.049); gastric pain 29% to 7% vs. 26% to 17% (*p* = 0.049); distention 79% to 43% vs. 61% to 31% (*p* = 0.283); constipation 21% to 11% vs. 13% to 4% (*p* = 0.66); anal pain 25% to 7% vs. 17% to 9% (*p* = 0.455)	None reported
*Punica granatum*	Kamali et al., 2015 [43]	Iran	Moderate UC	78	Placebo syrup	4 mL syrup containing 6 g dry pomegranate peel PO BID	Concomitant 5-ASA, immunosuppressive and steroids. Prednisolone > 15 mg/day, anti-TNFα agents, cyclosporine excluded.	10 weeks	(1) Clinical response: 48.3% vs. 36.4% (*p* = 0.441) (2) Change in symptoms from baseline: Improvement in fecal incontinence (*p* = 0.031) and general well-being (*p* = 0.013) in int. group; improvement in general well-being (*p* = 0.004) in cont. group.	No difference in mild AEs (urticaria, nausea, increased appetite). No serious AEs reported, but 2 int. and 1 cont. discontinued for UC flare.
Rose oil	Tavakoli et al., 2019 [44]	Iran	Moderate to severe UC	40	1000 mg liquid paraffin capsules PO TID before meals	1000 mg rose oil capsules PO TID before meals	Allowed to take concomitant medications	2 months	(1) Change in partial Mayo score: 3.93 ± 2.24 to 2.14 ± 1.61 (*p* = 0.022) vs. 3.86 ± 1.46 to 2.14 ± 1.46 (*p* = 0.014); *p* = 1 between groups (2) Change in IBDQ-9 scores: 41.6 ± 9.5 to 47.5 ± 8.3 (*p* = 0.03) vs. 44.6 ± 9.4 to 48.9 ± 6.5 (*p* = 0.012); *p* = 0.617 between groups (3) Change in fecal calprotectin: 64.21 ± 93.47 to 34.75 ± 89.45 (*p* = 0.229) vs. 67.56 ± 138.19 to 33.45 ± 2.72 (*p* = 0.122)	No difference in mild AEs (gastrointestinal side effects)
Saffron	Heydarian et al., 2022 [45]	Iran	Mild to moderate UC	80	Placebo tablets	100 mg saffron tablet PO QD	5-ASA, mesalamine, or azathioprine	8 weeks	(1) Change in mean ESR (mm/h): 15.40 ± 15.07 to 13.60 ± 14.32 (*p* = 0.002) vs. 13.91 ± 14.86 to 13.11 ± 11.23 (*p* = 0.622); *p* = 0.097 for difference in change between groups (2) Change in mean hs-CRP (µg/mL): 4.95 ± 2.03 to 3.76 ± 1.93 (*p* < 0.001) vs. 4.48 ± 1.95 to 4.56 ± 1.90 (*p* = 0.613); *p* = 0.001 for difference in change between groups (3) Change in mean TNFα (pg/mL): 30.51 ± 8.54 to 26.82. ± 7.50 (*p* < 0.001) vs. 31.80 ± 8.92 to 30.73 ± 8.17 (*p* = 0.187); *p* = 0.012 for difference in change between groups (4) Change in mean IBDQ9 score: 43.98 ± 7.39 to 45.33 ± 7.54 (*p* = 0.013) vs. 40.77 ± 10.53 to 40.69 ± 9.61 (*p* = 0.973); *p* = 0.068 for difference in change between groups	None reported
	Tahvilian et al., 2021 [46]	Iran	Mild to moderate UC	80	Placebo tablets	100 mg saffron tablet PO QD	5-ASA, mesalamine, or azathioprine	8 weeks	(1) Change from baseline SCCAI: −0.82 ± 1.05 vs. −0.02 ± 1.31 (*p* = 0.004) (2) Change in total antioxidant capacity (TAC) (nmol/mL): 0.11 ± 0.69 vs. −0.09 ± 0.39 (*p* = 0.016)	None reported
*Thymus kotschyanus*	Vazirian et al., 2022 [47]	Iran	Mild to moderate UC	50	Placebo capsules	500 mg *T. kotschyanus* PO in three divided doses daily	Concomitant mesalazine; all other concomitant medications were excluded	3 months	(1) Fecal calprotectin: 65.66 mg/kg + 37.42 vs. 145.06 mg/kg + 119.87 (*p* = 0.02) (2) SCCAIQ: median 6 vs. 7 (*p* = 0.015) (3) SIBDQ: median 43 vs. 39 (*p* = 0.329) (4) Seo Index: 109.77 + 21.32 vs. 109.94 + 17.94 (*p* = 0.981)	No difference in mild AEs (mouth ulcers, bloating)
Wheat grass	Ben-Arye et al., 2002 [48]	Israel	Colonoscopy with findings of active UC involving the left colon	24	100 mL placebo juice PO QD	100 mL wheat grass juice PO QD	5-aminosalicylic acid, prednisone	1 month	(1) Rectal bleeding (*p* = 0.025), abdominal pain (*p* = 0.019), DAI (*p* = 0.031), and PGA (*p* = 0.031) were significantly improved in int. vs. cont. (2) Sigmoidoscopic improvement: 78% vs. 30% (*p* = 0.13)	Mild AEs included nausea, decreased appetite, constipation
*Zingiber officinale*	Nikkhah-Bodaghi et al., 2019 [49]	Iran	Mild to moderate UC	64	Placebo capsules	Two 500 mg dried ginger powder capsules PO BID with meals (2000 mg total)	Not reported	12 weeks	(1) SCCAIQ: 7.6 ± 4.03 to 4.05 ± 1.23 (*p* = 0.438) vs. 6.2 ± 3.22 to 5.55 ± 2.39 (*p* = 0.194); *p* = 0.017 in between groups (2) IBDQ: 44.22 ± 9.79 to 47.23 ± 9.24 (*p* = 0.134) vs. 43.12 ± 6 to 41.87 ± 14.18 (*p* = 0.636); *p* = 0.14 in between groups (3) MDA: 8.33 ± 1.82 to 3.87 ± 1.95 (*p* < 0.001) vs. 7.88 ± 2.24 to 6.38 ± 2.42 (*p* = 0.119); *p* < 0.001 between groups	None reported

Abbreviations: PO (per oral), PR (per rectum), cont. (control), int. (intervention), SCCAI (Simple Clinical Colitis Activity Index), SCCAIQ (Simple Clinical Colitis Activity Index Questionnaire), IBDQ-9 (Inflammatory Bowel Disease Questionnaire-9), BID (twice daily), TID (three times daily), g/d (grams/day), AZA (azathioprine), 6-MP (6-mercaptopurine), AEs (adverse events), WBC (white blood cell), PSQI (Pittsburgh Sleep Quality Index), SIBDQ (Short Inflammatory Bowel Disease Questionnaire), OR (odds ratio), GSRS (Gastrointestinal Symptom Rating Scale), MDA (malondialdehyde).

### 3.1. Curcuma longa

Curcumin is an active polyphenol derived from the *Curcuma longa* rhizome, which is a member of the ginger family [15,20,25]. It has been used for centuries in Traditional Chinese Medicine (TCM) and Ayurveda and is designated as Generally Recognized as Safe (GRAS) as a food additive for cooking by the United States (US) Food and Drug Administration (FDA) [15,20,25]. Several laboratory and murine colitis model studies have established the antioxidant, antimicrobial, anti-inflammatory, and anticarcinogenic effects of curcumin [50,51,52,53,54,55,56,57]. The key anti-inflammatory mechanisms include the downregulation of the nuclear factor kappa-light-chain-enhancer of activated B cells (NF-κB) and other signaling pathways that play a central role in the pathogenesis of UC by increasing pro-inflammatory cytokine transcription and breaking down the intestinal barrier [58]. Curcumin has also been found to enhance intestinal barrier function through the upregulation of tight junction proteins, increased antioxidant enzyme activity, and the alteration of the microbiome with an increase in anti-inflammatory short-chain fatty-acid-producing bacteria [59,60,61]. 

#### 3.1.1. Clinical Evidence 

Our literature search identified eight RCTs investigating treatment with curcumin in active UC (Table 1). 

Four studies investigating oral curcumin supported its use as an added therapy to induce clinical remission in active UC. Lang et al. [15] reported that treatment with 3 g/day of oral curcumin resulted in significantly higher rates of clinical remission, clinical improvement, and endoscopic response compared to placebo in patients with mild to moderate UC taking mesalamine [15]. Similarly, Banerjee et al. [20] reported significantly higher rates of clinical response, clinical remission, and endoscopic remission among patients taking 50 mg/day of bioenhanced curcumin twice daily compared with placebo in patients taking mesalamine [20]. Masoodi et al. [24] studied curcuminoid nanomicelles with mesalamine and reported a reduction in the urgency of defecation score, mean Simple Colitis Clinical Activity Index (SCCAI) score, and patients’ self-reported well-being, although it was unclear how long patients had been taking oral mesalamine if at all prior to study onset [24]. Sadeghi et al. [25] reported higher rates of clinical response and clinical remission, decreased hs-CRP and ESR levels, and improved IBDQ-9 scores in patients taking oral curcumin compared to placebo in patients taking stable doses of UC medications, although a significant difference in education level between groups may have been confounding [25].

An enema formulation of curcumin in combination with oral 5-ASA for the treatment of ulcerative proctitis/procotsigmoiditis was investigated by Singla et al. [27]. This trial reported significantly higher rates of clinical response, clinical remission, and mucosal healing for the curcumin group compared to placebo in the per protocol analysis, but not the intention to treat analysis, which was attributed to a small sample size and high dropout rate in both groups [27]. 

Despite these promising results, there have been some studies with contradictory findings. Two abstracts with limited study details published by the same group reported data from studies investigating oral curcumin powder in combination with mesalamine or steroids [22,26]. Kumar et al. [22] reported that the proportion of patients who had a ≥25 point decrease in fecal calprotectin was significantly greater in the treatment group, although the difference in clinical response was not statistically significant when compared to placebo [22]. Shivakumar et al. [26] reported a statistical difference in the mean reduction in fecal calprotectin levels and a better, although not statistically significant, response in histologic activity, stool frequency, stool consistency, rectal bleeding, and Mayo score when compared to placebo [26]. The authors were not able to be contacted to inquire if the reported data in the two studies were from the same trial, but different clinical scales were used and different data were reported, suggesting separate study populations. Given that different outcomes were reported, data from the two abstracts were not pooled together in meta-analyses. 

Kedia et al. [21] also did not report a significant effect of oral purified curcumin capsules (450 mg/day) in combination with mesalamine on clinical response, clinical remission, or mucosal healing when compared to placebo, which was attributed to a potentially inadequate dose of curcumin [21].

**Figure 2:**
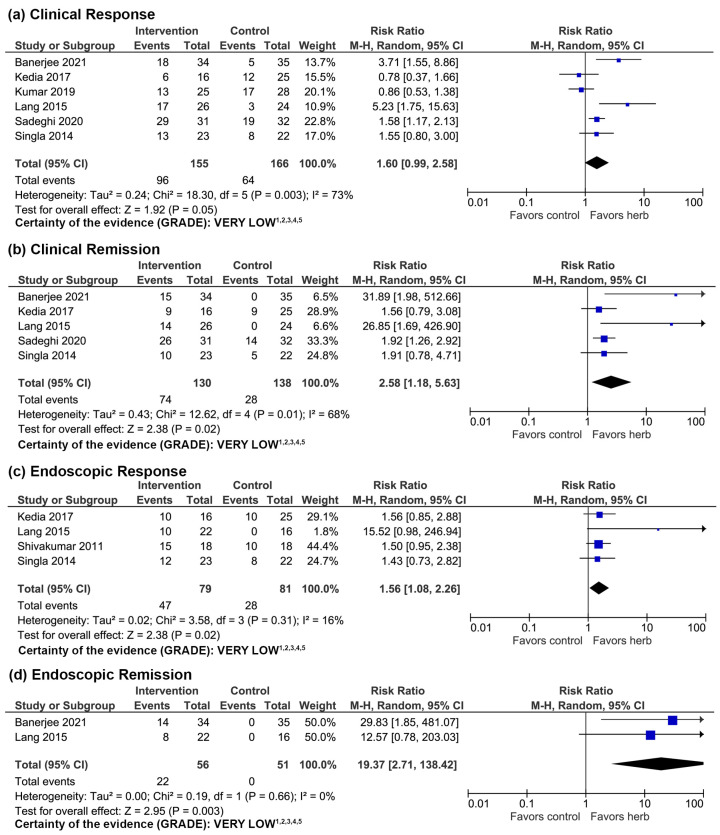
Meta-analyses with Forest plots of *Curcuma longa* for (**a**) clinical response, (**b**) clinical remission, (**c**) endoscopic response, and (**d**) endoscopic remission. CI = confidence interval. Certainty of evidence was downgraded for ^1^ risk of bias, ^2^ imprecision, ^3^ inconsistency, ^4^ indirectness, and ^5^ publication bias. CI = confidence interval.

Meta-analyses included 321 subjects from 6 RCTs for clinical response, 268 subjects from 5 RCTs for clinical remission, 160 subjects from 4 RCTs for endoscopic response, and 107 subjects from 2 RCTs for endoscopic remission. The rates of clinical remission (RR 2.58, 95% CI 1.18–5.63), endoscopic response (RR 1.56, 95% CI 1.08–2.26), and endoscopic remission (RR 19.37, 95% CI 2.71–138.42) were significantly greater with curcumin compared to control (Figure 2), suggesting efficacy in treating active UC. The difference in the rate of clinical response in the curcumin and control groups was not significant (RR 1.60, 95% CI 0.99–2.58). 

#### 3.1.2. Adverse Events

Serious adverse events (AEs) were only reported in three RCTs and primarily included subjects who were withdrawn from the study due to worsening UC [20,23,27]. However, the difference in incidence of mild or serious AEs was not statistically significant between treatment and control groups in any of the included studies. Mild adverse events included abdominal bloating, nausea, yellow stool, headaches, heartburn, and dyspepsia, among others. 

#### 3.1.3. Quality of Evidence 

The certainty of evidence using GRADE was very low (Figure 2). Study limitations included small sample sizes, heterogeneity in dosages and formulations between RCTs, and some RCTs only being published as an abstract with limited information available to assess quality of evidence (Table 2). 

### 3.2. Indigo naturalis

*Indigo naturalis* (IN), also known as Qing-Dai, is a dry pigment extract derived from a variety of plants, such as *Indigofera tinctoria*, and is traditionally used in TCM for inflammatory disorders, including UC [28,62]. The anti-inflammatory, antioxidant, and mucosal protection effects of IN have been attributed to the normalization of NFκB and mitogen-activated protein kinase (MAPK) expression, Th1 and Th2-mediated immune regulation, downregulation of inflammatory cytokine (IL-6, IL-8, TNF-α) production, attenuation of reactive oxygen species (ROS) and nitric oxide (NO) production, and facilitation of intestinal repair [63,64,65,66]. 

#### 3.2.1. Clinical Evidence

**Figure 3:**
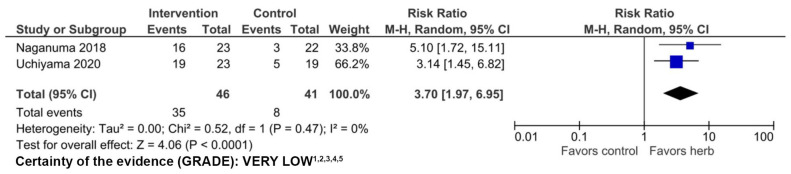
Meta-analyses with Forest plot of clinical response in *Indigo naturalis*. Certainty of evidence was downgraded for ^1^ risk of bias, ^2^ imprecision, ^3^ inconsistency, ^4^ indirectness, and ^5^ publication bias. CI = confidence interval.

Naganuma et al. [28] performed a multicenter study in Japan, in which patients with moderately active UC on conventional therapy were randomized to receive placebo or 0.5 g, 1 g, or 2 g IN daily for 8 weeks [28]. They reported a statistically significant and dose-dependent linear trend in clinical response rates and a significantly greater clinical remission rate in the 1 g and 2 g groups compared to placebo [28]. In another multicenter study, Uchiyama et al. [29] reported a significantly greater clinical response rate in patients with mild to moderate UC treated with 500 mg IN daily compared to placebo [29]. In pooled analysis of the 87 total subjects in these 2 RCTs, the clinical response rate was significantly greater (RR 3.70, 95% CI 1.97–6.95) among patients treated with 500 mg IN compared to placebo (Figure 3) suggesting efficacy in the treatment of active UC.

Although our study only identified two RCTs, there have been small observational and uncontrolled open-label studies that have further supported the efficacy of IN. Matsuno et al. [67] treated 33 moderate to severely active UC patients with 2 g of oral IN daily for one year with clinical remission rates of 67% and 73% and mucosal healing rates of 48% and 70% at weeks 4 and 52, respectively [67]. In a retrospective observational study by the same group, clinical response and remission rates at 4 weeks were 94.1% and 88.2%, respectively, in 17 active UC patients treated with 2–3 g/day of oral IN [68]. A pilot study of 20 patients with moderately active UC taking IN 2 g daily for 8 weeks had clinical response, clinical remission, and mucosal healing rates of 72%, 33%, and 61%, respectively [62]. In an open-label study of 11 patients with treatment refractory UC treated with 500 mg/day or 1.5 g/day oral IN, 10 patients achieved clinical response, all patients experienced endoscopic improvement, and 3 patients achieved clinical remission at 8 weeks [69]. A pilot study of 10 patients with active UC who were treated with 50 mg IN suppository for 4 weeks reported clinical remission and mucosal healing rates of 30% and 40%, respectively [70]. These studies, in addition to our meta-analysis findings, suggest that IN has a promising role in the adjuvant treatment of active UC. However, further investigation, particularly pertaining to safety, is required before IN can be recommended. 

#### 3.2.2. Adverse Events

Minor adverse events reported in the RCTs identified in this study included liver dysfunction, headache, epigastric/abdominal pain, nausea, palpitations, and constipation. However, the trial by Naganuma et al. [28] was terminated early because of an external report of pulmonary hypertension in a patient who used self-purchased IN for 6 months.

#### 3.2.3. Quality of Evidence

The certainty of evidence using GRADE was very low (Figure 3). Study limitations included possible selective reporting, a high attrition rate, and small sample sizes.

### 3.3. Andrographis paniculata

*Andrographis paniculata* is an herb used in Ayurveda and TCM. Its medicinal properties are attributed to andrographolides and other phytochemicals, which are anti-inflammatory and anti-microbial [71]. In active bacterial infections, andrographolides are found to decrease the proinflammatory expression of MAPK and inhibit NO production [71,72]. Andrographolides also inhibit platelet activating factor (PAF)-induced platelet aggregation in human polymorphonuclear leukocytes, which can have anti-inflammatory effects [71].

#### 3.3.1. Clinical Evidence

Sandborn et al. [30] investigated the use of an *A. paniculata* ethanol extract (HMPL-004) at doses of 1200 mg and 1800 mg daily in patients with UC taking mesalamine [30]. They reported significantly greater clinical response rates in the 1800 mg HMPL-004 group, but not the 1200 mg group when compared to the control. Mucosal healing rates were not significantly different between groups [30]. Similarly, Tang et al. [31] reported that clinical response, clinical remission, and endoscopic response rates were not statistically different between patients treated with 1200 mg/day of HMPL-004 compared to mesalazine [31]. 

**Figure 4:**
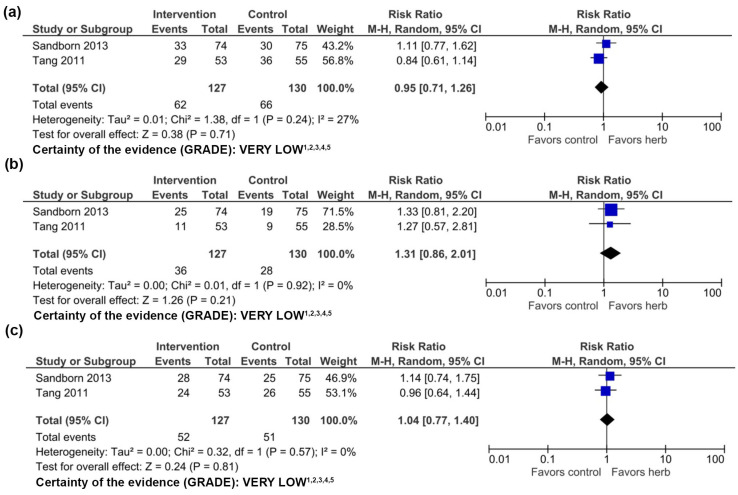
Meta-analyses and Forest plots of (**a**) clinical response, (**b**) clinical remission, and (**c**) endoscopic response in *Andrographis paniculata*. Certainty of evidence was downgraded for ^1^ risk of bias, ^2^ imprecision, ^3^ inconsistency, ^4^ indirectness, and ^5^ publication bias. CI = confidence interval.

A meta-analysis compiling data of 257 subjects treated with 1200 mg/day HMPL-004 compared to mesalamine or placebo with mesalamine was conducted. We found no statistically significant differences between groups for clinical response (RR 0.95, 95% CI 0.71–1.26), clinical remission (RR 1.31 95% CI 0.86–2.01), or endoscopic response (RR 1.04, 95% CI 0.77–1.40) (Figure 4).

#### 3.3.2. Adverse Events 

Both RCTs reported mild to moderate adverse events, including abdominal pain, dyspepsia, nausea, and flatulence in both groups. Tang et al. [31] reported that 4% of the treatment group had serious adverse events, including worsening UC, requiring hospitalization, and pregnancy, compared to 0% in the control group [31]. Sandborn et al. [30] reported a similar rate of minor adverse events across all groups, but reported a higher incidence of rash in the intervention groups [30]. 

#### 3.3.3. Quality of Evidence

The certainty of evidence using GRADE was very low (Figure 4). One potential issue with compiling this data is that the RCT by Tang et al. [31] compares mesalazine to HMPL-004 alone, while patients treated with HMPL-004 in the RCT by Sandborn et al. [30] were permitted to be taking concomitant mesalazine. Sandborn et al. [30] reported a significantly greater clinical response rate in patients taking 1800 mg HMPL-004 with mesalazine compared to mesalazine only, suggesting that higher doses may be more effective [30]. Neither study mentioned limitations, suggesting a high reporting bias. Further research is needed to make definitive conclusions.

### 3.4. Aloe vera

The mucilaginous gel from the leaf pulp of *Aloe vera* (*Aloe barbadensis* Miller), a perennial succulent, has been used medicinally in Indian, Chinese, Egyptian, and European cultures to treat gastrointestinal conditions, skin injuries, osteoporosis, and cancer [32,73]. *Aloe vera*’s antioxidant and anti-inflammatory properties have been attributed primarily to its anthraquinones, including aloe-emodin and its c-glycoside, aloin, which possess peroxyl radical scavenging activity [74]. *Aloe vera* is thought to enhance the intestinal barrier function by inducing mucin expression and increasing the thickness of the mucus layer, which can mitigate inflammation and colonic tissue damage [75].

#### 3.4.1. Clinical Evidence

Langmead et al. [32] compared oral *Aloe vera* gel with placebo in a 2:1 ratio among patients with mild to moderate UC [32]. Although median SCCAI and histologic scores decreased in the treatment group, the rates of clinical improvement, clinical remission, and sigmoidoscopic remission were not statistically significant [32]. Similarly, a small study by Pica et al. [33] revealed a significant decrease in the average disease activity index (DAI) score among 10 patients treated with daily *Aloe vera* gel enemas in combination with oral mesalamine, which was not seen in the placebo group [33]. 

#### 3.4.2. Adverse Events 

Serious adverse events were not reported in Langmead et al. [32] and were not discussed in the abstract by Pica et al. [32,33]. However, given the small size of the available studies, definitive conclusions about safety and efficacy cannot be made. 

#### 3.4.3. Quality of Evidence

The overall quality of evidence is low given limited data. Limitations of the study by Langmead et al. [32] include having a small sample size, a 20–21% attrition rate, inter-observer variability for sigmoidoscopic scoring, and a variety of baseline concomitant UC medications, although these factors were balanced among groups. Only an abstract was available to evaluate the study by Pica et al. [33], so evaluation of risk of bias was limited and limited outcome data were reported, suggesting a higher risk of reporting bias. 

### 3.5. Arthrospira platenesis

*Arthrospira platensis*, also known as spirulina, is a blue–green microalgae with antioxidant properties that is a natural source of vitamins (vitamins B12 and provitamin A), minerals, and phytochemicals (carotenoids and phycocyanins) [34]. A murine colitis study showed that treatment with spirulina reduced DSS-induced damage, reduced ROS, increased antioxidant enzyme activity, and modulated gut microbiota [56]. 

#### 3.5.1. Clinical Evidence

Moradi et al. [34] evaluated the effects of spirulina capsules on anthropometric indices, mood, sleep, and quality of life in patients with mild to moderate UC taking conventional therapy [34]. No significant effects were found on anthropometric indices such as body mass index (BMI) and blood pressure. Both treatment and control groups had a significant increase in quality of life and reduction in sleep disturbances and stress scores over the study period, but there was also a significant difference in quality of life and stress scores between groups at the end of the study [34]. 

#### 3.5.2. Quality of Evidence

This study had a low risk of bias but, overall, the quality of evidence for its use in UC is very low given the limited data. 

### 3.6. Boswellia serrata

*Boswellia serrata*, also known as Indian frankincense, is a plant grown in India, Northern Africa, and the Middle East [76]. Although it is widely used as incense in cultural and ceremonial settings, its gum resin extracts have also been used to treat inflammatory diseases. This is due to the anti-inflammatory activity of boswellic acids, such as acetyl-11-keto-ß-boswellic acid, which specifically inhibits a key enzyme in leukotriene synthesis, 5-lipoxygenase [35,76]. *B. serrata* has also been shown to inhibit MAPKs as well as TNF-α and NO production in human peripheral mononuclear cells [76]. 

#### 3.6.1. Clinical Evidence

Gupta et al. [35] investigated the use of Sallai gugal (*B. serrata*) capsules compared to sulfasalazine in eight patients with grade II-III UC in an open non-randomized trial [35]. Both groups had similar rates of clinical remission and experienced an improvement in symptoms, such as abdominal pain and mucus, blood, or necrotic material in the stool. Although outcomes were intentionally not statistically compared between groups, similarity in outcomes may suggest non-inferiority between *B. serrata* and sulfasalazine [35]. 

#### 3.6.2. Adverse Events

In total, 17.6% of the intervention group reported mild adverse effects, including nausea, abdomen fullness, and epigastric pain, compared with 0% of the control group [35]. 

#### 3.6.3. Quality of Evidence 

The quality of evidence in active UC is very low due to limited data. The study by Gupta et al. [35] was not randomized and had a small sample size, with a disproportionate intervention to control ratio, selective reporting, and unclear study outcomes, resulting in a high risk of bias. *B. serrata* has been studied in other forms of IBD, such as Crohn’s disease (CD) and collagenous colitis, as well as experimental colitis models with mixed results, suggesting that further investigation is warranted to clarify its potential role in the treatment of UC [77]. 

### 3.7. Green Tea

Green tea (*Camellia sinensis*) leaves are rich in polyphenols including catechins, such as epigallocatechin-3-gallate (EGCG), which are thought to have antioxidant, anti-inflammatory, and anticarcinogenic effects [36,78,79]. The anti-inflammatory effects of EGCG have been attributed to the inhibition of NF-κB, the downregulation of inflammatory cytokines, and the upregulation of tumor growth factor ß (TGFß) expression [36,79,80,81,82].

#### 3.7.1. Clinical Evidence

In a trial by Dryden et al. [36], participants with mild to moderate UC were randomized in a 4:1 ratio to low dose oral EGCG-rich polyphenol E 200 mg twice daily, high dose polyphenol E 400 mg twice daily, or placebo [36]. Clinical response and clinical remission rates were 66.7% and 53.3%, respectively, in the combined intervention group and 0% in the placebo group. 

#### 3.7.2. Adverse Events

No serious adverse events were reported, although one patient in the intervention group required hospitalization for *C. difficile* infection [36]. 

#### 3.7.3. Quality of Evidence

Given the small sample size and inadequate powering of the study by Dryden et al., definitive conclusions cannot be made about the safety or efficacy of polyphenol E in active UC. However, multiple murine colitis model studies have shown promising results that should encourage further large scale RCTs given the accessibility and tolerability of green tea [80]. 

### 3.8. Flaxseed

Flax (*Linum usitatissimum*) is a plant that is cultivated as a food crop and is thought to be a functional food due to its soluble fiber, α-linolenic acid (ALA), and phytoestrogen content [38]. The oil derived from flax seeds is rich in antioxidants, polyphenols, and omega-3 polyunsaturated fatty acids (PUFAs), including ALA, which is a precursor to eicosapentaenoic acid (EPA). A study of colon mucosa biopsies from UC patients found that inflamed mucosa has higher levels of arachidonic acid and lower levels of ALA and EPA compared to control. Omega-3 PUFAs competitively inhibit the conversion of arachidonic acid to inflammatory eicosanoids (e.g., prostaglandins, leukotrienes) and activate the anti-inflammatory transcription peroxisome proliferator activator receptor (PPAR)-γ [83]. Animal colitis models have also reported that ALA decreases inflammatory cytokine production, NFκB activation, and intestinal permeability [84,85]. 

#### 3.8.1. Clinical Evidence

In an RCT by Morshedzadeh et al. [37,38], patients with mild to moderate UC taking 5-ASAs were randomized to ground flaxseed (GF), flaxseed oil (FO), or a control group that only received medical advice [37,38]. After 12 weeks, there were significant decreases in fecal calprotectin, Mayo score, ESR (*p* < 0.001), INF-γ (*p* < 0.001), IL-6 (*p* < 0.001), waist circumference (*p* = 0.02), diastolic blood pressure (DBP) (*p* < 0.001), and systolic blood pressure (SBP) (*p* < 0.001), as well as a significant increase in TGF-β (*p* < 0.001) and Inflammatory Bowel Disease Questionnaire-9 (IBDQ-9) score in the GF and FO groups compared to control [37]. There was no statistical difference between the FO and GF groups except for a greater increase in TGF-β in the GF group (*p* = 0.007) [37]. 

#### 3.8.2. Adverse Events

Adverse events were not reported in the articles by Morshedzadeh et al. [37,38].

#### 3.8.3. Quality of Evidence 

These findings suggest potential for improving clinical response rates in UC, but further research is needed. The current evidence for the use of flaxseed is very low given the limited number of studies and high risk of bias. Limitations of the study by Morshedzadeh et al. [37,38] were its open-label design and lack of an appropriate placebo in the control group.

### 3.9. Licorice 

Licorice is an herbaceous flowering plant that has been used in Chinese, Indian, and Greek medicine for centuries. The active compounds in licorice include triterpinoids, such as glycyrrhizin, and flavonoids, such as glabiridin [86]. Licorice has been found to suppress inflammatory cytokine and ROS production, inducible nitric oxide synthase (iNOS), myeloperoxidase (MPO), cyclooxygenase (COX), and NFκB, resulting in anti-inflammatory and anti-oxidant effects [86]. 

#### 3.9.1. Clinical Evidence

Sun et al. randomized patients with active UC to treatment with oral mesalazine or a combination of oral licorice decoction with mesalazine [39]. Clinical total effective rate, IL-10 levels, and IBDQ scores were significantly higher (*p* < 0.05) while interleukin-6 (IL-6), IL-17, and tumor necrosis factor-α (TNF-α) levels were significantly lower (*p* < 0.05) in the licorice group compared to control after 6 weeks [39]. 

#### 3.9.2. Adverse Events

No adverse events were reported in the limited information available in the abstract by Sun et al. [39].

#### 3.9.3. Quality of Evidence

Only an abstract for this published study was available in English, so risk of bias was difficult to adequately ascertain. The quality of evidence for licorice as a treatment of active UC is, therefore, very low. 

### 3.10. Olive Oil

Extra virgin olive oil (EVOO) is a central component of the Mediterranean diet and contains beneficial polyphenols, including oleuropein, hydroxytyrosol, oleocanthal, and flavonoids, which are thought to have antioxidant, anti-inflammatory, and anticancer effects [87]. Olive oil polyphenols scavenge ROS, decrease iNOS expression, decrease angiogenesis, and decrease inflammation by downregulating PPARγ and NFκB activation, as well as inflammatory cytokine production [87,88]. Treatment of human colon mucosa biopsies from patients with UC with oleuropein resulted in decreased inflammatory infiltrate, disappearance of focal cryptitis, and recovery of goblet cells [88].

#### 3.10.1. Clinical Evidence

Morvaridi et al. [40] studied the effect of EVOO consumption in patients with UC with both active and in remission disease on conventional therapy with a single-blind randomized crossover trial [40]. Subjects received 20 days of 50 mL daily EVOO followed by 14 days of washout and then 20 days of 50 mL daily canola oil (CO) or 20 days of CO followed by 14 days of washout and 20 days of EVOO. Although the change in Mayo score was not significant, bloating, constipation, fecal urgency, and incomplete defecation scores as assessed by the Gastrointestinal Symptom Rating Scale as well as ESR and hs-CRP levels significantly decreased after EVOO consumption compared to CO [40]. 

#### 3.10.2. Adverse Events

No significant adverse events were reported in the study by Morvaridi et al. [40]. 

#### 3.10.3. Quality of Evidence

The overall quality of evidence for the use of EVOO in UC is very low given the limited number of clinical trials with high risk of bias. Sources of bias in the study by Morvaridi et al. [40] include the subjects not being blinded to the intervention, the use of per protocol analysis, and the failure to disclose the proportion of subjects in remission vs. active disease. Although several laboratory and animal studies have elucidated the potential benefits of plant-derived polyphenols in EVOO [87], further research in UC should be encouraged given its accessibility, tolerability, and role in the Mediterranean diet. 

### 3.11. Pistacia lentiscus

*Pistacia lentiscus* (mastiha) is an evergreen shrub native to the Mediterranean that is cultivated for its aromatic resin, mastic gum, and has been recognized by the European Medicines Agency for treating mild dyspepsia and wounds [41]. *P. lentiscus* is rich in triterpenoids, which exert antioxidant effects by increasing intracellular antioxidant glutathione and anti-inflammatory effects through the downregulation of NFκB [89,90]. 

#### 3.11.1. Clinical Evidence 

Papada et al. [41] investigated the use of oral *P. lentiscus* tablets in patients with active UC on conventional therapy for 3 months [41]. There was a significant improvement in IBDQ scores (*p* = 0.004) and significant decreases in fecal lysozyme (*p* = 0.018) and fibrinogen (*p* = 0.006) levels in the treatment group, while fecal lactoferrin (*p* = 0.001) and calprotectin (*p* = 0.029) levels increased in the placebo group. There was no significant difference in oxidative stress markers between groups. The trial investigated amino acid (AA) profiles given a potential link between AA profiles and IBD pathogenesis, and reported that allo-isoleucine, isoleucine, lysine, tyrosine, and tryptophan levels significantly decreased in the placebo group over time, but remained largely unchanged in the treatment group, with the exception of increased tyrosine levels [41].

#### 3.11.2. Adverse Events

No adverse events were reported in the study by Papada et al. [41]. 

#### 3.11.3. Quality of Evidence

The risk of bias of this RCT was low, but the overall level of evidence is very low given the limited data available to suggest improvement in biomarkers of intestinal inflammation and quality of life in patients with UC. 

### 3.12. Plantago major

*Plantago major*, also known as broadleaf plantain, is a member of the plantain family that is widely distributed around the world and has a variety of therapeutic uses in Traditional Persian Medicine (TPM) [91,92]. Its bioactive compounds, including flavonoids, alkaloids, and triterpenoids, among others, are thought to have anti-inflammatory, antioxidant, anticancer, anti-diarrheal, and wound healing properties [91,92,93].

#### 3.12.1. Clinical Evidence

Baghizadeh et al. [42] evaluated treatment with 3600 mg/day of roasted *P. major* seed capsules compared to roasted wheat flour control capsules on gastrointestinal symptoms in patients with UC (active or remission) on conventional therapy [42]. Abdominal tenderness (*p* = 0.011), gastroesophageal reflux (*p* = 0.049), and gastric pain (*p* = 0.049) significantly decreased in the treatment group compared to control. Although there was no significant difference in visible blood in the stool between the two groups, the treatment group experienced a significant decrease in visible blood compared to baseline after 8 weeks (*p* = 0.001), while the control group exhibited no significant improvement (*p* = 0.331). Diarrhea, nocturnal diarrhea, fecal incontinence, abdominal pain, abdominal distension, anal pain, and well-being were not significantly different between groups at 8 weeks [42]. 

#### 3.12.2. Adverse Events

No adverse events were reported in the study by Baghizadeh et al. [42]. 

#### 3.12.3. Quality of Evidence

The quality of evidence is very low given the limited data available to suggest that *P. major* in addition to conventional therapy may help alleviate some gastrointestinal symptoms in patients with UC. 

### 3.13. Punica granatum

Pomegranate (*Punica granatum*) is a fruiting shrub native to the Middle East and India and has been used medicinally for inflammation, ulcers, and diarrhea [43]. Pomegranate peel is rich in polyphenols and its anti-inflammatory, antioxidant, anticancer, and wound healing potential has been attributed to flavonoids, hydrolyzable tannins, and metabolic products of ellagitannins [43,94,95]. Pomegranate peel has been found to increase antioxidant enzymes, regulate the gut microbiome, and downregulate iNOS, COX-2, MAPK, NFκB, and inflammatory cytokine production [95,96].

#### 3.13.1. Clinical Evidence 

Kamali et al. [43] investigated the use of 8 mL pomegranate peel extract containing 6 g of pomegranate peel daily compared to placebo in patients with moderate UC taking conventional therapy [43]. They found that the rate of clinical response was significantly higher in the treatment group compared to placebo at week 4 (41.4% vs. 18.2%, *p* = 0.055), but not at week 10 (48.3% vs. 36.4%, *p* = 0.441). Both groups had significant reductions in mean Lichtiger Colitis Activity Index (LCAI) scores. At week 10, there was a significant improvement in fecal incontinence (*p* = 0.031) and general well-being (*p* = 0.013) in the treatment group, while there was only an improvement in general well-being (*p* = 0.004) in the placebo group [43]. 

#### 3.13.2. Adverse Events

Adverse events were mild (i.e., urticaria, nausea, increased appetite) and not significantly different between groups in the study by Kamali et al. [43].

#### 3.13.3. Quality of Evidence

These promising results suggest that pomegranate peel may have potential for improving clinical response rates as an added therapy. However, the overall quality of evidence is low. Limitations of the study by Kamali et al. include a small sample size, possibly resulting in an underpowered study and the use of a per protocol analysis, although it was noted that those who discontinued the study were not significantly different with regard to demographics or symptoms when compared with those remaining in the study.

### 3.14. Rose Oil

Rose oil is produced by soaking rose petals, mainly *Rosa damascena* (damask rose) and *Rosa centifolia* (cabbage rose), in carrier oils such as sesame oil [44]. Damask rose petals contain flavonoids, polyphenols, vitamin C, and the monoterpene geraniol, which have anti-inflammatory and anti-oxidant effects, lending to its use in TPM for a variety of ailments including digestive disorders [44,97]. In a murine colitis model, oral geraniol administration reduced disease activity index (DAI), improved stool consistency, decreased inflammatory cytokine and MPO activity in colon cells, demonstrated the downregulation of NFκB, iNOS, and COX-2, and increased glutathione and superoxide dismutase (SOD) activity [98].

#### 3.14.1. Clinical Evidence

Tavakoli et al. [44] evaluated the use of rose oil capsules in patients with moderate to severe UC on conventional therapy [44]. Partial Mayo and IBDQ-9 scores decreased significantly in both groups over time, but there was no significant difference between groups [44]. There was a non-significant decrease in fecal calprotectin in both groups [44]. 

#### 3.14.2. Adverse Events

There was no difference in mild gastrointestinal side effects between groups in the study by Tavakoli et al. [44].

#### 3.14.3. Quality of Evidence

The high attrition rate (30%) is a limitation of the study by Tavakoli et al. [44], with 10% and 15% in the treatment and control groups, respectively, withdrawing from the study due to gastrointestinal discomfort. The study showed no significant difference in outcomes between treatment and control groups at the end of the study period. The evidence for the use of rose oil in UC is very low.

### 3.15. Saffron

Saffron is a culinary spice cultivated from the stigma of *Crocus sativus* flowers, which are grown in the Mediterranean and Asia. Saffron is thought to have several therapeutic applications with memory-improving, anti-depressant, anti-inflammatory, and anti-tumor effects, which are attributed to the major bioactive compounds including the carotenoids crocetin, crocin, safranal, and picrocrocin [45,46]. The proposed antioxidant and anti-inflammatory mechanisms of these compounds include increasing glutathione, antioxidant enzyme, and nuclear factor erythroid 2-related factor 2 (Nrf2) activity, as well as decreasing iNOS, NFκB, and inflammatory cytokine expression [45,99]. Murine colitis models have suggested that crocin can ameliorate wound healing and inflammation in chemically induced colitis and suppress colitis-associated carcinogenesis [45,99]. 

#### 3.15.1. Clinical Evidence 

We identified one RCT that investigated the effects of oral saffron on oxidative stress in mild to moderate UC, with outcome data presented in two articles [45,46]. The treatment group had a significantly greater reduction in mean SCCAI scores and a significantly greater increase in SOD, total antioxidant capacity (TAC), and glutathione levels compared to control at 8 weeks [46]. Significant decreases in TNFα and hs-CRP levels, as well as an increase in IL-10 (*p* = 0.004) levels were reported in the treatment group compared to control [45]. There were no differences in ESR, IL-17, and IBDQ-9 between groups, although there were significant decreases in ESR (*p* = 0.02) and IL-17 (*p* = 0.001), as well as an increase in IBDQ-9 (*p* = 0.013) in the treatment group after 8 weeks [45]. 

#### 3.15.2. Adverse Events

There were no adverse events reported in the included studies. Saffron is thought to be safe at doses of 1.5 g/day, although doses greater than 5 g/kg/day are considered toxic [46]. 

#### 3.15.3. Quality of Evidence

Current available evidence is limited, making the quality of evidence low. However, these promising results suggest that saffron as an added therapy can improve mean SCCAI scores and inflammatory and antioxidant markers in UC. In addition, the accessibility and safety of saffron as a culinary spice should encourage further investigation for the potential use of saffron in UC. 

### 3.16. Thymus kotschyanus

*Thymus kotschyanus* is an aromatic perennial herb native to the Mediterranean, of which the aerial parts have been used in TPM for a variety of ailments due to its antispasmodic, antibacterial, and antioxidant effects [47,100]. The oil components, including thymol, carvacol, and geraniol, among others, contain flavonoids, polyphenols, anthocyanins, and other bioactive polyphenols [47]. The monoterpene thymol has exhibited anti-inflammatory, antioxidant, and antitumor potential through free radical scavenging [101], cyto/genotoxic effects on colorectal cancer cells [102], NFκB and MAPK inhibition, and downregulation of COX-2 expression, inflammatory cytokine production, and NO production in murine colitis models and in vitro studies [47,103].

#### 3.16.1. Clinical Evidence

Vazirian et al. [47] investigated the use of *Thymus kotschyanus* capsules in patients with mild to moderate UC on stable doses of mesalamine [47]. At 12 weeks, the treatment group had a significantly lower mean fecal calprotectin level and median SCCAI score when compared to placebo, while there was no difference in median SIBDQ score, Seo index, ESR (*p* = 0.572), or CRP (*p* = 0.160) [47]. 

#### 3.16.2. Adverse Events

Only mild adverse events, including mouth ulcers and bloating, were reported, with no significant difference between groups in the study by Vazirian et al. [47]. No serious adverse events were reported. 

#### 3.16.3. Quality of Evidence

There was a significant baseline difference in mean SIBDQ score between groups at the outset of the study by Vazirian et al. [47], which may have influenced outcomes. Other limitations of this study include a small sample size, attrition rate, and lack of clarity if a per protocol or intention to treat approach was used to analyze outcomes. 

A variety of plants, including thyme and oregano, contain thymol, carvacrol, and thyme polyphenols. Thyme and oregano are regarded as safe with negligible toxicity by the US Food and Drug Administration [102]. Although the quality of the existing evidence for treatment in UC is very low, further investigation of plants containing thyme oil may be beneficial given the accessibility, general safety and tolerability, and promising results from laboratory and animal studies. 

### 3.17. Wheatgrass

Wheatgrass is the sprouted leaves of the wheat plant, *Triticum aestivum*, which is grown in North America and Europe and has been used in indigenous healing [104]. Its therapeutic qualities have been attributed to chlorophyll, vitamins C and E, and flavonoids, such as apigenin, which have anticarcinogenic, anti-inflammatory, and antioxidant effects [48,104,105].

#### 3.17.1. Clinical Evidence

Ben-Ayre et al. [48] investigated the use of wheatgrass juice in UC patients with sigmoidoscopic evidence of left colon involvement [48]. They reported significant differences in rectal bleeding, abdominal pain, DAI score, physician global assessment, and patients’ retrospective evaluation (*p* = 0.0053) in the treatment group compared to placebo [48]. Sigmoidoscopic improvement was not statistically significant between groups, although 7/9 subjects in the treatment group showed improvement, compared to 3/10 in the placebo group [48]. 

#### 3.17.2. Adverse Events

Mild reported adverse events in the study by Ben-Ayre et al. [48] included nausea, decreased appetite, and constipation [48]. 

#### 3.17.3. Quality of Evidence

A limitation of the study by Ben-Arye et al. [48] was that 6/11 and 2/12 patients in the treatment and placebo groups, respectively, believed they were getting wheatgrass, suggesting that participant blinding may not have been effective. Although the risk of bias was low in this RCT, the overall quality of evidence is very low given limited available data. 

### 3.18. Zingiber officinale

*Zingiber officinale*, or ginger, has been prevalent in herbal medicine for centuries and used for various conditions in Ayurveda, TCM, and TPM [106]. The therapeutic qualities of ginger can be attributed to its bioactive terpene and phenolic compounds, such as gingerols, zingiberene, and shogaols [106]. Murine colitis models and in vitro studies suggest that the anti-inflammatory and antioxidant activities of ginger are due to the inhibition of NFκB, increased production of anti-inflammatory cytokines, decreased production of inflammatory cytokines, and increased levels of SOD and glutathione [106]. Ginger has also been found to have antimicrobial and anticarciniogenic effects [106] and can modulate the gut microbiome by increasing short chain fatty acid (SCFA)-producing bacteria, which are thought to be anti-inflammatory [107]. 

#### 3.18.1. Clinical Evidence

Nikkhah-Bodaghi et al. [49] studied the use of 2000 mg ginger powder capsules in two divided doses with meals daily vs. placebo in 64 patients with mild to moderate UC [49]. Average SCCAI score was significantly decreased in the ginger group compared to placebo at 12 weeks [49]. Although there was no difference in TAC, malondialdehyde (MDA) levels were significantly decreased in the ginger group and compared to placebo at 12 weeks [49].

#### 3.18.2. Adverse Events

There were no adverse events reported in the study by Nikkhah-Bodaghi et al. [49]. 

#### 3.18.3. Quality of Evidence 

While the risk of bias of this RCT was determined to be low, it is difficult to make definitive conclusions on the effects of ginger on UC given the small number of studies available. However, given that ginger is accessible, prevalent in cooking, and generally tolerable, further research should be encouraged to expand on these promising findings suggesting that ginger can improve SCCAI scores and antioxidant markers in UC. 

## 4. Discussion

Herbal medicines are being used with increasing frequency by patients with UC, due to continued relapsing disease despite treatment, the perception that herbal remedies are less toxic than current standards of care, and the increasing availability of herbal supplements. Despite this, healthcare providers often have limited knowledge or skills in guiding their patients regarding the safety and efficacy of herbal products. This systematic review compiles data and provides a comprehensive resource of RCTs for herbal medicines used in the treatment of active UC to inform future research directions, treatment guidelines, and help healthcare providers guide patients in their use. 

There were 28 RCTs investigating 18 different herbs for the treatment of active UC identified in our literature search. *Curcuma longa*, otherwise known as curcumin, was the most studied and can be recommended as an added therapy to induce clinical remission in UC. Our meta-analysis revealed improved rates of clinical remission, endoscopic response, and endoscopic remission when compared to control, but we did not find a significant difference in clinical response rates. This may be due to the inclusion of studies by Kumar et al. [22], which had a higher risk of bias and lower quality compared to other studies due to only an abstract being available, and Kedia et al. [21], which had a high attrition rate and lower dose, in the meta-analysis for clinical response. Pooled data should be interpreted with caution given that the sample size despite pooling studies remained <300, there was moderate heterogeneity among studies evaluated for clinical response and remission, and the overall quality of the evidence was very low. However, there are notably promising preclinical and clinical results that should encourage further investigation with higher quality and larger scale RCTs using consistent and accessible formulations and doses. Given its favorable safety profile, curcumin can likely be recommended as an adjunctive treatment in addition to the standard of care for inducing remission in active UC.

*Indigo naturalis* has potential benefits and is used extensively in TCM. The pooled sample size from two RCTs in our meta-analysis was small (*n* = 81) and the quality of evidence was very low. However, we found a significantly improved rate of clinical response in the treatment group compared to placebo. In addition, there have been small observational and uncontrolled open-label studies that have supported the efficacy of IN [67,68,69,70]. However, adverse events associated with IN have been reported with case reports of pulmonary arterial hypertension, intussusception, and ischemic colitis [108,109,110,111]. A Japanese nationwide survey of 877 UC patients using IN reported liver dysfunction (*n* = 40), gastrointestinal symptoms (*n* = 21), headache (*n* = 13), and PAH (*n* = 11), although liver dysfunction and PAH were reversible after discontinuation of IN and no IN-associated deaths were reported [28]. Until higher quality and larger scale studies can verify the safety of IN, we recommend that its use should be approached with caution. 

We did not find a statistical difference in rates of clinical response, clinical remission, or endoscopic response between patients treated with 1200 mg daily of oral HMPL-004 (*A. paniculata*) or placebo in a pooled analysis of 257 subjects. However, the RCT by Sandborn et al. [30] found that a higher dose of 1800 mg daily of oral HMPL-004 had a significantly higher rate of clinical response when compared to placebo (*p* = 0.018), with no dose-dependent toxicity [30]. Although the recommended dose of *A. paniculata* extract in the Chinese Pharmacopoeia is listed as 0.63–1.26 g/day, this suggests that further research with higher doses of *A. paniculata* may yield more promising results. With regard to safety, the incidence of adverse events was similar among treatment and control groups although a reversible mild rash was reported in 8% of patients receiving *A. paniculata* vs. 1% in the control group in the RCT by Sandborn et al. [30,31]. 

The single RCTs identified for *Boswellia serrata*, green tea, and *Punica granatum* revealed a statistically significant improvement in clinical response and/or clinical remission and would benefit from further investigation. Improvement in disease activity scores observed with treatment with saffron, *Thymus kotschyanus*, or wheatgrass suggests that they could potentially elicit a clinical response. Studies of *Plantago major*, olive oil, and wheatgrass reported improvement in symptoms of UC, such as abdominal pain or blood in stools. The use of *Arthrospira platensis*, flaxseed, licorice, and *Pistacia lentiscus* was found to improve quality of life in patients with active UC. Some RCTs also reported improvement in inflammatory markers, such as hs-CRP, ESR, IL-6, and TNFα, when treated with licorice, flaxseed, saffron, or *Thymus kotschyanus*. Many of the studies were conducted on patients taking 5-ASAs or other conventional UC treatments, suggesting a potential role for herbal medicines as an added therapy to improve clinical response and clinical remission rates in UC that warrants further investigation with larger scale RCTs. 

Despite these promising findings, the quality of evidence was determined to be very low for most herbs due to the small number of RCTs available and small sample sizes. Four of the studies included were only available as abstracts, which limited available information to assess the quality of evidence. The included studies had variable risk of bias (Table 2). Many of the studies excluded patients with medical comorbidities, which may limit the generalizability of the findings. The majority of studies have looked at herbal medicines as an added therapy to conventional treatment, which limits their potential use to this setting. The herbal medicines discussed here are not covered by insurance and their associated costs can be a barrier to their use.

With regard to safety, few serious adverse events were reported and the incidence of adverse events was comparable between treatment and control groups in the majority of RCTs discussed. However, larger scale studies are needed to verify safety. Many herbal constituents have been noted to interact with CYP450 enzymes in vitro and may result in theoretical herb–drug interactions [112], although further in vivo studies are needed. In addition, the FDA classifies herbal medicines as food supplements and they are, therefore, not subject to the FDA’s rigorous drug approval process imposed on pharmaceutical drugs [112]. Although the FDA has implemented rules for Good Manufacturing Practices, there is limited enforcement and significant variability in manufacturing practices, resulting in a lack of biological and pharmaceutical equivalence between products. This limits the reliability of over-the-counter herbal supplements and many of the specific formulations used in the discussed studies are not commercially available in the United States.

## 5. Conclusions

Our study has several strengths including a rigorous methodology for systematic review and meta-analysis, a focus on RCTs to present the highest quality evidence available, and a comprehensive review including 18 different herbal medicines. However, our study is not without limitations. To provide a focused review of the treatment of active UC, we did not include studies of patients in remission, who may also benefit from herbal medicines. This review does not include preclinical, in vitro, pilot, observational, and other study designs, which may have limited the amount of evidence identified in our literature search. While our study focuses on individual herbs, we recognize that this is not inclusive of combination herbal formulations which are likely more representative of the traditional healing practices from which they originated. Many of these herbs have been traditionally used in countries outside of the United States or Europe, so a greater body of literature may be published in languages other than English and were, unfortunately, not able to be included in our review.

Given the prevalence of herbal supplement use in the US, it is imperative that healthcare providers are educated on how to guide their patients in safe herbal medicine use. Providers should be aware of resources to vet herbal supplements, such as the Natural Medicines Comprehensive Database available online or the National Institute of Health’s HerbList smartphone application. It is also crucial for patients and providers to be familiar with herbal supplement companies that have appropriate manufacturing practices and are transparent in their compound sourcing, ingredients, and techniques to verify potency and test for contaminants. More stringent federal and industry-led regulatory practices should be encouraged to ensure the safe production and use of herbal medicines.

We identified several herbs with the potential to expand the armamentarium of treatment options in UC. The promising results highlighted in this review and the growing popularity of herbal supplement use necessitates investment in research with larger scale and higher quality RCTs. As we begin to develop a larger body of evidence obtained with rigorous methodology, we can gain confidence in the efficacy and safety of herbal medicines and develop meaningful evidence-based recommendations for their use in the treatment of ulcerative colitis. 

## Figures and Tables

**Table 2:** Risk of bias of articles identified in systematic review.

Herb	Author/Year	Random Sequence Generation (Selection Bias)	Allocation Concealment (Selection Bias)	Double Blinding of Participants and Researchers (Performance Bias)	Blinding of Outcome Assessment (Detection Bias)	Incomplete Outcome Data (Attrition Bias)	Selective Reporting (Reporting Bias)	Other Bias	Comments
*Curcuma longa*	Banerjee et al., 2021 [20]	Low	Unclear	Low	Unclear	Low	Low	Low	Limited information regarding allocation concealment.
	Kedia et al., 2017 [21]	Low	Low	Low	Unclear	High	Low	Low	High attrition rate in both groups, resulting in uneven number of participants completing study.
	Kumar et al., 2019 [22]	Low	Unclear	Low	High	High	Unclear	Unclear	Only abstract available with limited details. Small sample size and did not specify how many participants in each group.
	Lang et al., 2015 [23]	Low	Low	Low	Unclear	Low	Low	Low	Limited information regarding blinding of outcome concealment.
	Masoodi et al., 2018 [24]	Low	Low	Low	Unclear	Low	Low	Low	Limited information regarding blinding of outcome assessment.
	Sadeghi et al., 2020 [25]	Low	Low	Low	Unclear	Low	Low	Low	Limited information regarding blinding of outcome assessment.
	Shivakumar et al., 2011 [26]	Unclear	Unclear	Low	Unclear	Unclear	Low	High	Only abstract available with limited details.
	Singla et al., 2014 [27]	Low	Low	Low	Unclear	Low	High	Low	Limited information regarding blinding of outcome assessment. States that adverse events were documented, but are not described.
*Indigo naturalis*	Naganuma et al., 2018 [28]	Low	Low	Low	Low	High	Low	Low	Many patients in placebo group discontinued study resulting in lower clinical response rate. Trial terminated early due to external reasons.
	Uchiyama et al., 2020 [29]	Low	Unclear	Unclear	Unclear	Low	Low	Low	Blinding and allocation concealment not discussed.
*Andrographis paniculata*	Sandborn et al., 2013 [30]	Low	Low	Low	Unclear	Low	High	Low	Block randomization schedule was utilized; however, it is not specified whether outcome assessment was performed with blinding. Did not specify adverse events that resulted in 16 people discontinuing study drug. Limitations not mentioned.
	Tang et al., 2013 [31]	Low	Low	Low	Low	High	High	Low	≥20% attrition rate in placebo group. Limitations not mentioned.
*Aloe vera*	Langmead et al., 2004 [32]	Low	Low	Low	Low	High	High	Low	≥20% attrition rate in both groups; reported change in histologic score and SCCAI as statistically significant, although not a clear outcome.
	Pica et al., 2021 [33]	High	High	Low	Unclear	High	Unclear	Unclear	Only abstract available. Reported 44 study participants, but only reported data for 14 participants. Study outcomes not clear.
*Arthrospira platensis*	Moradi et al., 2021 [34]	Low	Low	Low	Low	Low	Low	Low	Per protocol analysis used.
*Boswellia serrata*	Gupta et al., 1997 [35]	High	Unclear	High	Unclear	High	High	High	Reported 50 study participants and no dropouts but only reported data for 42 participants. Non-randomized study drug allocation. Small sample size with >4:1 intervention–placebo ratio. Study outcomes unclear. Likely inadequately powered.
Green tea	Dryden et al., 2013 [36]	Low	Low	Low	Unclear	High	Low	High	Small sample size with 4:1 intervention–placebo ratio; likely inadequately powered.
Flaxseed	Morshedzadeh et al., 2019 [37]	Low	High	High	Unclear	Low	Low	Low	No allocation concealment or double blinding because this was an open-label study.
	Morshedzadeh et al., 2021 [38]	Low	High	High	Unclear	Low	Low	Low	No allocation concealment or double blinding because this was an open-label study.
Licorice	Sun et al., 2018 [39]	Unclear	Unclear	Low	Unclear	Unclear	High	Unclear	Only abstract available, so limited information provided. Only *p*-values reported.
Olive oil	Morivaridi et al., 2020 [40]	Low	High	High	Low	High	Low	Low	Single-blinded crossover trial. No discussion of subject blinding. Carryover and period effects reported. Proportion of disease activity states (remission vs. active disease) not reported. Per protocol analysis used.
*Pistacia lentiscus*	Papada et al., 2018 [41]	Low	Low	Low	Unclear	Low	Low	Unclear	Small sample size and unclear whether outcomes were blinded.
*Plantago major*	Baghizadeh et al., 2021 [42]	Low	Low	Low	Low	Unclear	Low	Low	Small sample size and moderate attrition rate in both groups.
*Punica granatum*	Kamali et al., 2015 [43]	Low	Low	Low	Low	Unclear	Low	Low	Uneven attrition rate and use of per protocol analysis, although it is reported that those who discontinued the study were not significantly different regarding demographics or symptoms when compared to those who completed the study.
Rose oil	Tavakoli et al., 2019 [44]	Low	Low	Low	Low	High	Low	Low	High attrition rate (30%).
Saffron	Heydarian et al., 2022 [45]	Low	Low	Low	Unclear	Low	Low	Unclear	Unclear whether outcome assessors were blinded.
	Tahvilian et al., 2020 [46]	Low	Low	Low	Unclear	Low	Low	Unclear	Unclear whether outcome assessors were blinded.
*Thymus kotschyanus*	Vazirian et al., 2022 [47]	Low	Low	Low	Unclear	High	High	High	Uneven attrition rate after randomization. Unclear if intention to treat or per protocol analysis used. Significant baseline difference in SIBDQ may influence outcomes.
Wheat grass	Ben-Arye et al., 2002 [48]	Low	Low	Unclear	Low	Low	Low	Low	6/11 patients receiving wheatgrass believed they were receiving wheatgrass, while 2/12 patients receiving placebo believed they were receiving wheatgrass which raises concern regarding blinding of participants.
*Zingiber officinale*	Nikkhah-Bodaghi et al., 2019 [49]	Low	Low	Low	Unclear	Low	Low	Low	Unclear whether outcome assessors were blinded. No significant differences in baseline characteristics between both groups.

## Data Availability

The data underlying this article are included in this published article. Data can be shared upon reasonable request to the corresponding author.

## Supplementary Materials

The following supporting information can be downloaded at: https://www.mdpi.com/article/10.3390/nu16070934/s1. PRISMA 2020 Checklist. Reference [113] is cited in the supplementary materials.
